# Risk Factors for Acute Respiratory Distress Syndrome in Sepsis Caused by Peptic Ulcer Perforation: A Retrospective Study

**DOI:** 10.3390/diagnostics16142258

**Published:** 2026-07-20

**Authors:** Yavuz Selim Kahraman, Veysel Garani Soylu, Öztürk Taşkın, Ufuk Demir

**Affiliations:** 1 Department of General Surgery, Division of Surgical Oncology, Kastamonu Training and Research Hospital, Kastamonu 37150, Türkiye; 2 Department of Intensive Care, Kastamonu University, Kastamonu 37150, Türkiye; vgsoylu@kastamonu.edu.tr; 3 Department of Anesthesiology and Reanimation, Kastamonu University, Kastamonu 37150, Türkiye; otaskin@kastamonu.edu.tr (Ö.T.); udemir@kastamonu.edu.tr (U.D.)

**Keywords:** acute respiratory distress syndrome, peptic ulcer perforation, sepsis, intra-abdominal infection

## Abstract

**Background/Objectives:** Our study aimed to identify risk factors for the development of ARDS (acute respiratory distress syndrome) in patients with sepsis resulting from peptic ulcer perforation. **Methods:** Between January 2018 and December 2025, patients aged 18 years and older who underwent open surgery for peptic ulcer perforation and were diagnosed with sepsis due to intra-abdominal infection related to peptic ulcer perforation during postoperative follow-up or hospitalization were included in the study. Daily ARDS screening was performed within 7 days of sepsis diagnosis. Patients were divided into groups with and without ARDS. Laboratory values were recorded within the first 24 h after sepsis diagnosis, and APACHE II score, SOFA score, NEWS, and SAPS II were calculated based on these values and the patient’s clinical condition. **Results:** The SOFA score showed the highest discriminative ability, with an AUC of 0.710 (95% CI: 0.619–0.794) at a cutoff of 9.0, representing 54.2% sensitivity and 83.1% specificity. Albumin level showed an AUC of 0.654 (95% CI: 0.560–0.748) at a cutoff of 2.40 g/dL; its specificity was high (85.1%), but its sensitivity was relatively low (41.7%). CAR showed moderate predictive performance with an AUC of 0.627 (95% CI: 0.522–0.730) at a cutoff of 61.11, representing 60.4% sensitivity and 72.1% specificity. The combined model demonstrated the best predictive performance with an AUC of 0.727 (95% CI: 0.635–0.820), 70.8% sensitivity, and 72.7% specificity. **Conclusions**: SOFA score demonstrated the best overall clinical applicability for early ARDS risk stratification, while both SOFA score and APACHE II score showed independent associations with ARDS in separate multivariable models.

## 1. Introduction

Peptic ulcer disease remains a widespread health problem today, with a prevalence ranging from 1.5% to 3% [1]. With the introduction of proton pump inhibitors into clinical practice, a significant decrease in the incidence of peptic ulcers has been observed, and the incidence of peptic ulcer perforation has declined in parallel [2]. However, perforation remains the most common indication requiring surgical intervention in patients with peptic ulcers. Surgical mortality rates in cases of perforated peptic ulcers range from 10% to 40%, and these rates remain high [3,4]. Sepsis is one of the key factors influencing mortality in patients with perforated peptic ulcers. It has been reported that approximately 30–35% of patients present with sepsis at hospital admission, and sepsis is responsible for 40–50% of deaths related to peptic ulcer perforation [5].

Acute respiratory distress syndrome (ARDS) is a clinical condition characterized by widespread inflammatory injury to the lung parenchyma [6]. Various risk factors, including lung-origin or extrapulmonary infections, shock, trauma, and aspiration, contribute to the etiology of ARDS [7]. From a pathophysiological perspective, ARDS is explained by two primary mechanisms: the first involves the leakage of protein-rich fluid into the alveolar space due to alveolar epithelial damage, and the second involves fluid leakage from the capillary endothelium into the pulmonary interstitium due to systemic inflammation. In direct ARDS, alveolar epithelial damage is predominant, whereas in indirect ARDS, capillary endothelial damage is dominant [8].

Intra-abdominal sepsis refers to the systemic inflammatory response developed by the host organism in response to intra-abdominal infections. While appendicitis and cholecystitis are among the primary causes of intra-abdominal infections, peptic ulcer perforations are also significant etiological factors. In sepsis resulting from intra-abdominal infections, the lungs are the most commonly affected target organ, and this condition frequently leads to the development of ARDS. Indeed, sepsis is the most common extrapulmonary cause of ARDS [9,10].

ARDS is a serious clinical condition with high mortality rates, and the prognosis is even worse in cases secondary to intra-abdominal infections [11]. Therefore, identifying risk factors for ARDS development in cases of sepsis resulting from intra-abdominal infections and developing preventive strategies to address them are of great importance for improving patient prognosis.

In recent years, only a limited number of studies have examined the development of ARDS following sepsis associated with intra-abdominal infections [12,13]. However, these studies generally encompass all causes of intra-abdominal infections rather than focusing on a specific source of infection. The literature regarding the development of ARDS following sepsis associated with peptic ulcer perforation consists solely of case reports [14]. Furthermore, no study has yet comprehensively evaluated the risk factors for the development of ARDS in this patient group.

Although clinical severity scores and routinely available laboratory biomarkers have been determined in various sepsis populations, their predictive value in patients with sepsis due to peptic ulcer perforation has not been specifically investigated. Therefore, the present study addresses a significant gap in the existing literature by focusing on the disease-specific clinical application of these readily available parameters to predict ARDS in this single-center population.

## 2. Materials and Methods

### 2.1. Study Design and Setting

This retrospective observational study was conducted in patients who underwent open surgery for peptic ulcer perforation and developed sepsis due to intra-abdominal infection. Ethical approval for the study was obtained from the Kastamonu University Faculty of Medicine Committee on Non-Interventional Research (date: 19 March 2026, approval number: 2026-68). The study was conducted in accordance with the principles of the Declaration of Helsinki (1964).

### 2.2. Study Population

Patients aged 18 years and older who underwent open surgery for peptic ulcer perforation between January 2018 and December 2025 were included. Patients diagnosed with sepsis caused by intra-abdominal infection related to peptic ulcer perforation during postoperative follow-up or at the time of hospital admission were identified using the hospital information management system. Because the study exclusively enrolled patients with sepsis secondary to peptic ulcer perforation, the source of intra-abdominal infection was uniform across the entire study cohort.

### 2.3. Inclusion Criteria and Exclusion Criteria

**Figure 1:**
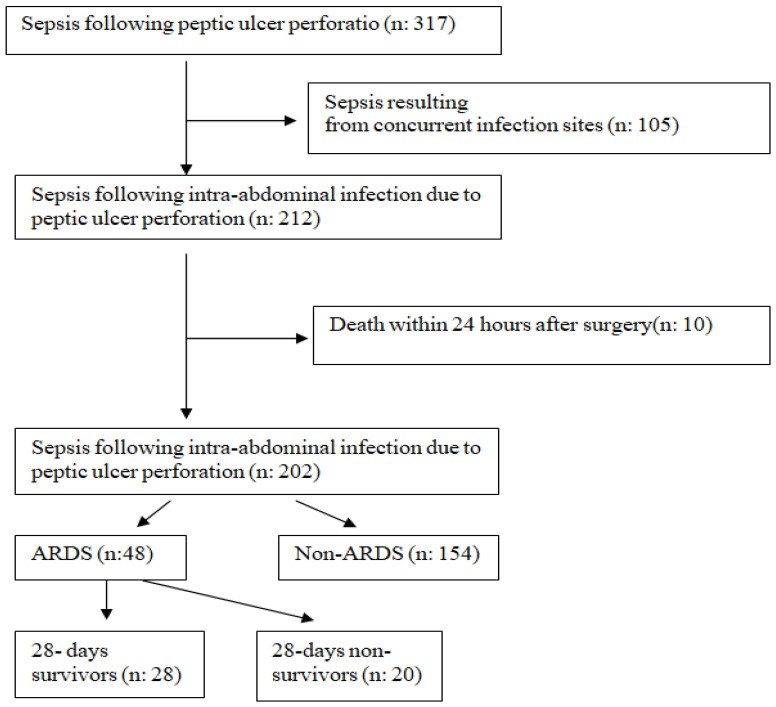
Flowchart of patient inclusion.

The diagnosis of intra-abdominal infection due to peptic ulcer perforation was based on clinical parameters such as abdominal pain, tenderness, and systemic inflammatory signs (fever, elevated white blood cell count, tachycardia, and tachypnea), as well as radiological criteria [15]. The diagnosis of sepsis was made according to the Sepsis 3.0 criteria [16]. Patients with missing clinical data or those who did not receive standardized management were excluded from the study. No missing data were present in the final study dataset after application of the exclusion criteria. Patients who died within the first 24 h after surgery (*n* = 10) were excluded from the study. Appropriate standard clinical treatment was defined according to the recommendations of the Surviving Sepsis Campaign guidelines and institutional management protocols. These include prompt source control, early administration of broad-spectrum antibiotics, adequate fluid resuscitation, vasopressor support when indicated, and organ-supportive intensive care management. During the patient enrollment process, four observers evaluated whether patients received appropriate standard clinical treatment in accordance with treatment recommendations established by the Surviving Sepsis Campaign guidelines; data were recorded for patients who received appropriate standard clinical treatment [17]. Data extraction was performed according to predefined study criteria using the institutional electronic medical record system to maximize data consistency and minimize information bias. Any discrepancies among the four reviewers regarding patient eligibility or compliance with the predefined treatment criteria were resolved by consensus after joint review of the complete medical records. The exclusion criteria for the study were: sepsis resulting from infections at other concurrent sites, sepsis caused by intra-abdominal infection due to a perforated peptic ulcer, and death occurring within 24 h post-surgery. Patients who died within the first 24 h after surgery were excluded because ARDS diagnosis according to the Berlin Definition requires adequate clinical observation and radiological evaluation during follow-up, which could not be reliably performed in these patients. The diagnosis of ARDS was made according to the Berlin Definition of ARDS, and ARDS screening was performed daily for 7 days following the diagnosis of sepsis; patients included in the study were divided into groups with and without ARDS [18]. A total of 317 patients who underwent surgery due to peptic ulcer perforation and received a diagnosis of sepsis were identified through the hospital information management system. In total, 105 patients were excluded from the study due to sepsis originating from concurrent infections in other sites, and 10 patients were excluded because they died within 24 h post-surgery. Following the exclusion criteria, 202 patients were included in the study. Of the 202 patients, 48 were in the group that developed ARDS within 7 days of sepsis, while 154 were in the group that did not develop ARDS (Figure 1).

### 2.4. Data Collection

Through a retrospective review of the hospital information management system, the age, sex, comorbidities, body mass index (BMI), and mortality status within 28 days post-surgery of the patients included in the study were recorded. Laboratory values within the first 24 h following the diagnosis of sepsis were recorded, and based on these values and the patient’s clinical condition, the acute physiology and chronic health evaluation II (APACHE) score, sequential organ failure assessment II (SOFA score), national early warning score (NEWS), and Simplified Acute Physiology Score II (SAPS) were calculated and recorded. Data on septic shock, vasopressor requirement, and other shock-related variables were not systematically available and were therefore not included in the analysis. Patients were divided into 4 groups according to the World Health Organization’s body mass index classification standard: underweight (BMI < 18.5 kg/m^2^), normal weight (BMI: 18.5–24.9 kg/m^2^), overweight (BMI: 25–30 kg/m^2^), and obese (BMI > 30 kg/m^2^).

### 2.5. Statistical Analysis

The statistical analyses for this study were performed using IBM SPSS Statistics, version 26.0 (IBM Corp., Armonk, NY, USA). The distribution of continuous variables was expressed as mean ± standard deviation (mean ± SD). Categorical variables were presented as counts and percentages [n (%)]. For comparisons between groups, the independent samples Student’s *t*-test was used for continuous variables. For comparisons of categorical variables, the chi-square test or, when necessary, Fisher’s exact test was applied. Variables found to be statistically significant in univariate analysis, together with clinically relevant covariates selected a priori, including disease severity, comorbidities, and demographic characteristics, were considered for inclusion in the multivariate logistic regression model to minimize the effects of potential confounding. Variables exhibiting multicollinearity were carefully evaluated, and only the SOFA score was retained among the disease severity indices because of its stronger clinical relevance and statistical performance. The initial multivariable model included sex, respiratory disease, albumin, total bilirubin, platelet count, CAR, lactate, and the SOFA score. Variables that were not statistically significant or did not improve overall model performance were excluded from the final model using backward stepwise selection. The final multivariable model included four predictor variables with 48 outcome events, corresponding to approximately 12 events per variable, which is considered adequate to reduce the risk of model overfitting. Results were reported as odds ratios (OR) along with 95% confidence intervals (95% CI). Model calibration was assessed using the Hosmer–Lemeshow goodness-of-fit test. A receiver operating characteristic (ROC) curve analysis was conducted to evaluate the predictive performance for ARDS. Model robustness was further evaluated using five-fold stratified cross-validation. Patients were randomly allocated into five approximately equal-sized folds. Stratification was based on ARDS status to preserve the proportion of patients with and without ARDS in each fold. Multicollinearity among predictor variables was assessed using the variance inflation factor (VIF) analysis, with VIF values <5 considered indicative of acceptable collinearity. The VIF values ranged from 1.13 to 1.40, indicating that no significant multicollinearity was detected. The area under the curve (AUC), sensitivity, specificity, and optimal cutoff values were calculated. Optimal cutoff values were determined using the Youden index. A *p*-value of <0.05 was considered statistically significant in all analyses.

## 3. Results

A total of 202 patients were included in the study; 48 of them (23.8%) developed ARDS, while 154 (76.2%) did not. There was no statistically significant difference in age between the groups (55.83 ± 10.99 vs. 54.49 ± 11.39 years, *p* = 0.502). Regarding gender, female patients were significantly more common in the ARDS group than in the non-ARDS group (64.6% vs. 35.7%, *p* < 0.001). Regarding comorbidities, respiratory disease was significantly more common in patients with ARDS (33.3% vs. 13.0%, *p* = 0.003); the presence of other comorbidities—diabetes mellitus, hypertension, cerebrovascular disease, cardiovascular disease, and kidney disease—was not statistically significant (all *p* > 0.05). The 28-day mortality rate in the ARDS group was statistically significantly higher than in the non-ARDS group (41.7% vs. 15.6%, *p* < 0.001). BMI was one of the statistically significant parameters between the groups (*p* = 0.010). Patients with BMI <18.5 kg/m^2^ and BMI >30 kg/m^2^ were proportionally more common in the ARDS group (for BMI <18.5 kg/m^2^: 6.5% vs. 18.8%; for BMI >30 kg/m^2^: 4.5% vs. 12.5%).

In laboratory analyses, albumin levels were significantly lower in patients with ARDS (2.66 ± 0.61 vs. 2.97 ± 0.52 g/dL, *p* = 0.002), and total bilirubin levels were significantly higher (1.16 ± 0.61 vs. 0.92 ± 0.50 mg/dL, *p* = 0.017). The inflammatory burden assessed by CAR was also significantly higher in the ARDS group and was statistically significant (50.53 ± 33.94 vs. 67.87 ± 43.88, *p* = 0.014). Platelet count was significantly lower in patients with ARDS (168.23 ± 91.56 vs. 202.83 ± 95.31 × 10^3^/μL, *p* = 0.026). No statistically significant differences were observed between the groups in terms of creatinine, CRP, white blood cell count, hemoglobin, hematocrit, neutrophil count, lymphocyte count, monocyte count, NLR, and lactate levels (all *p* > 0.05). In the comparison of scores between groups, APACHE II score, SAPS II, and SOFA score were significantly higher in the ARDS group compared to the non-ARDS group and were statistically significant (all *p* < 0.001). However, there was no statistically significant difference in NEWS between the groups (*p* = 0.766) (Table 1).

Multivariate logistic regression analysis was performed to identify independent predictors of acute respiratory distress syndrome. Variables found to be statistically significant in univariate analysis and clinically relevant were included in the multivariate logistic regression model. To avoid multicollinearity, the primary multivariable model initially included only one disease severity score (SOFA score). Additional multivariable logistic regression analyses were subsequently performed using APACHE II score and SAPS II as alternative disease severity indices while maintaining the same covariate structure. The SOFA score was an independent risk factor for ARDS (OR: 1.13, 95% CI: 1.01–1.26, *p* = 0.030). Female gender showed a borderline association with the development of ARDS (OR: 2.09, 95% CI: 0.99–4.41, *p* = 0.054). Albumin level and pre-existing respiratory disease were not independent risk factors for ARDS after adjusting for confounding factors (*p* > 0.05). (Table 2).

In the APACHE II score model, APACHE II score remained independently associated with ARDS (OR: 1.06, 95% CI: 1.00–1.13, *p* = 0.047). In the SAPS II model, SAPS II showed a borderline but non-significant association with ARDS (OR: 1.04, 95% CI: 0.99–1.08, *p* = 0.086). These findings were consistent with the primary SOFA score-based model.

**Figure 2:**
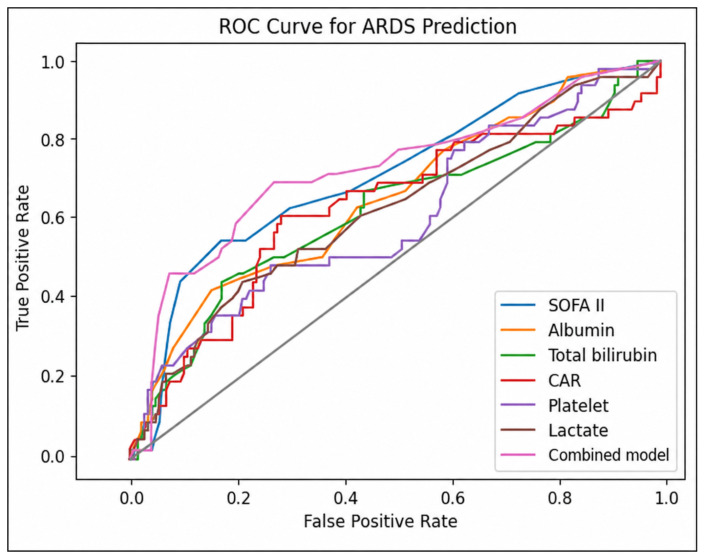
Receiver operating characteristic (ROC) curve analysis for predicting acute respiratory distress syndrome (ARDS). ROC, receiver operating characteristic; AUC, area under the curve.

The Hosmer–Lemeshow goodness-of-fit test showed good model calibration (χ^2^ = 8.26, df = 8, *p* = 0.409). A receiver operating characteristic curve analysis was performed to evaluate the predictive performance of key variables for the development of acute respiratory distress syndrome. Among individual parameters, the SOFA score demonstrated the highest discriminatory ability with an AUC value of 0.710 (95% CI: 0.619–0.794), a sensitivity of 54.2%, and a specificity of 83.1% at a cutoff value of 9.0. Albumin level showed an AUC value of 0.654 (95% CI: 0.560–0.748) at a cutoff value of 2.40 g/dL, with high specificity (85.1%) but relatively low sensitivity (41.7%). CAR demonstrated moderate predictive performance with an AUC value of 0.627 (95% CI: 0.522–0.730), a sensitivity of 60.4%, and a specificity of 72.1% at a cutoff value of 61.11. Total bilirubin, platelet count, and lactate levels demonstrated limited discriminatory ability with AUC values ranging from 0.606 to 0.624. A combined model created using all these parameters, however, demonstrated the best predictive performance with an AUC of 0.727 (95% CI: 0.635–0.820), 70.8% sensitivity, and 72.7% specificity (Table 3, Figure 2).

To assess the internal validity of the combined prediction model, five-fold stratified cross-validation was performed. The model demonstrated stable discriminatory performance, with a mean cross-validated AUC of 0.707 (SD 0.037), compared with an apparent AUC of 0.727 in the original dataset, indicating minimal overfitting and acceptable internal robustness.

**Figure 3:**
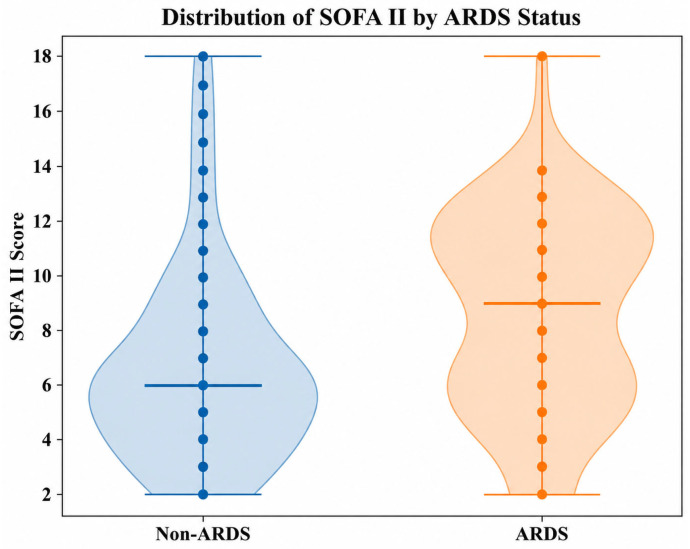
Distribution of the sequential organ failure assessment (SOFA) score in patients with or without acute respiratory distress syndrome (ARDS).

A violin plot was used to compare the distribution of SOFA scores between patients with and without acute respiratory distress syndrome. The plot shows that SOFA scores were distributed at higher values in patients who developed ARDS compared to the non-ARDS group. While scores in the non-ARDS group were predominantly clustered in the low-to-moderate range (approximately 3–6), the distribution in the ARDS group was concentrated in higher score ranges (particularly 9–12). According to the graph, the distribution in the ARDS group was broader and exhibited a bimodal distribution. In the non-ARDS group, however, the distribution was more compact and concentrated around lower scores (Figure 3).

## 4. Discussion

In this study, risk factors for ARDS were investigated in patients who developed sepsis following intra-abdominal infection due to peptic ulcer perforation. The main findings of our study were a high incidence of ARDS (23.8%), a significantly increased 28-day mortality rate in patients who developed ARDS, and the identification of a high SOFA score as an independent risk factor. Additionally, lower albumin levels and higher CAR values were associated with ARDS in univariate analyses; however, these variables did not remain independent predictors after multivariable adjustment. These results highlight the significant impact of systemic inflammation caused by intra-abdominal sepsis on pulmonary complications.

Intra-abdominal infections are one of the leading causes of sepsis and carry a high risk for the development of ARDS. In a large retrospective study published by Ma et al. in 2024, ARDS occurred in 29.5% of patients with sepsis due to intra-abdominal infection. Furthermore, this study demonstrated that intensive care and 28-day mortality rates were significantly increased in patients who developed ARDS [12]. The 23.8% ARDS rate identified in our study is consistent with these findings. However, the fact that our patient population consists solely of cases of sepsis due to peptic ulcer perforation places our study in a more unique position within the current literature. This is because most previous studies have included heterogeneous groups of intra-abdominal infections.

The significantly higher mortality rate observed in patients who developed ARDS is one of the most important findings of our study. The literature reports that mortality associated with sepsis-related ARDS remains high. Multicenter cohort studies and meta-analyses have demonstrated that the development of ARDS significantly increases both short-term and long-term mortality [19,20]. The finding that the mortality rate was approximately three times higher in the ARDS group in our study supports the critical impact of pulmonary complications on prognosis in intra-abdominal sepsis due to peptic ulcer perforation. In particular, the widespread peritoneal inflammation that develops following gastrointestinal perforation may be one of the underlying mechanisms of this relationship, as it increases cytokine release and leads to alveolocapillary membrane damage.

Systematic reviews and meta-analyses examining ARDS development in patients presenting with sepsis have reported that high SOFA scores and APACHE scores are strongly associated with the development of ARDS [21]. Moreno et al.’s study also demonstrated that disease severity scores were significantly higher in the group that developed ARDS [22]. In our study, the fact that the SOFA score had the highest AUC value in the ROC analysis indicates that the severity of organ dysfunction is a strong predictor of the development of pulmonary complications. In particular, the ease of application of the SOFA score suggests that this parameter could be used in clinical practice for early risk stratification. To address the Academic Editor’s recommendation, we additionally evaluated the APACHE II score and SAPS II in separate multivariable models. APACHE II score also remained independently associated with ARDS, whereas SAPS II showed a borderline but non-significant association. Although the predictive performances of the three models were broadly comparable, the SOFA score demonstrated the most favorable combination of clinical simplicity and discriminative ability, supporting its use as the preferred bedside risk stratification tool in this patient population.

In our study, low albumin levels were significantly more common in the ARDS group. Hypoalbuminemia is one of the key indicators of systemic inflammation and poor clinical status. A decrease in albumin levels may increase pulmonary interstitial fluid accumulation by lowering intravascular oncotic pressure and facilitating the development of alveolar edema. In their study, McNeil and colleagues noted a linear relationship between hypoalbuminemia and the risk of acute respiratory distress syndrome in critically ill adults [23]. However, in the multivariate analysis conducted in our study, albumin was not an independent risk factor. This finding can be explained by the fact that albumin levels reflect the overall severity of the illness rather than ARDS directly.

The finding that CAR levels were elevated in the ARDS group further supports the importance of the inflammatory response. While CRP is a positive acute-phase reactant, albumin is a negative acute-phase reactant; therefore, CAR is considered a more comprehensive marker of systemic inflammation. It is known that inflammatory cytokine activation plays a significant role in sepsis-associated ARDS. The literature emphasizes that septic ARDS is characterized by an intense inflammatory response, endothelial dysfunction, and immune dysregulation [24]. Our results also suggest that the increase in CAR levels may reflect this inflammatory burden.

The fact that the female gender showed a borderline significant association is consistent with conflicting results in the literature. Although some studies suggest that the inflammatory response is more pronounced in women, the exact mechanism of this association remains unclear. The absence of an independent association in our study may be due to the limited sample size.

Previous studies examining the relationship between gastrointestinal perforation and ARDS are generally case reports or small patient series. In particular, it has been reported that subphrenic inflammation following perforation may directly lead to ARDS via diaphragmatic spread and secondary lung injury [14,25]. However, there are very few studies that systematically examine ARDS risk factors in patients with sepsis due to peptic ulcer perforation. Therefore, our study contributes to the existing literature on ARDS in patients with sepsis secondary to peptic ulcer perforation.

The combined model developed in our study demonstrated higher predictive performance than individual biomarkers alone. In recent years, nomograms and combined risk models have been developed to predict the development of ARDS following gastrointestinal perforation [13]. However, during our literature review, we did not encounter any studies specifically focused on the group of patients who developed ARDS following sepsis due to peptic ulcer perforation. This finding suggests that combining multiple clinical and laboratory parameters to predict ARDS development may result in a better predictive model compared to individual biomarkers.

The present study contributes to the existing literature by evaluating established clinical severity scores and laboratory biomarkers in patients with sepsis secondary to peptic ulcer perforation. Although this study provides clinically significant evidence regarding early determinants of ARDS in patients with sepsis due to peptic ulcer perforation, further prospective multicenter studies using different methodological approaches are needed to strengthen the evidence in this area.

### 4.1. Limitations

This study has several limitations. First, owing to its retrospective, single-center observational design, causal relationships cannot be established, and the risks of selection and information bias cannot be completely eliminated. Because the study relied on routinely collected electronic medical records, inconsistencies or incomplete documentation may have affected the accuracy of some clinical variables. Furthermore, the exclusion of patients who died within the first 24 h after surgery may have introduced survivor bias, potentially leading to an underestimation of both disease severity and the true incidence of ARDS. In addition, only patients who underwent open surgery were included. Although this approach provided a relatively homogeneous surgical cohort and reduced treatment-related heterogeneity, it may limit the generalizability of our findings to patients undergoing laparoscopic surgery or non-operative management. Second, the relatively small sample size may have limited the statistical power to detect weaker associations. Third, several potentially important variables, including pro-inflammatory cytokines, detailed mechanical ventilation parameters, microbiological culture results, ICU length of stay, pre-admission medication use (e.g., corticosteroids or non-steroidal anti-inflammatory drugs), lifestyle-related factors (e.g., alcohol consumption), and long-term pulmonary outcomes, were unavailable for analysis. Although multivariable logistic regression was performed to adjust for clinically relevant confounding factors, including disease severity, comorbidities, and demographic characteristics, residual confounding inherent to retrospective observational studies cannot be completely excluded. Finally, prospective multicenter studies with larger patient populations are warranted to externally validate our findings and confirm their applicability across broader clinical settings.

### 4.2. Future Research Directions

Our study is primarily based on clinical and laboratory parameters. Translational and mechanistic studies investigating the effect of systemic inflammation on lung injury in intra-abdominal sepsis associated with peptic ulcer perforation may be planned in the future. Additionally, although it is known that sepsis-associated ARDS exhibits heterogeneous biological phenotypes, the presence of these phenotypes and their association with clinical outcomes in patients with sepsis due to peptic ulcer perforation could be a subject for further research.

## 5. Conclusions

In conclusion, ARDS is a frequent and serious complication of sepsis secondary to peptic ulcer perforation and is associated with substantially increased short-term mortality. Among the evaluated severity scores, both the SOFA score and APACHE II score demonstrated independent associations with ARDS in separate multivariable models, whereas SAPS II did not reach statistical significance. Considering its simplicity, bedside applicability, and comparable predictive performance, the SOFA score may represent the most practical tool for early risk stratification in this patient population. Although lower albumin levels and higher CAR values were associated with ARDS in univariate analyses, these markers did not remain independent predictors after adjustment and should therefore be interpreted as indicators of overall disease severity rather than causal risk factors. Importantly, the routine assessment of SOFA score together with readily available laboratory parameters during the first 24 h after sepsis diagnosis may facilitate early risk stratification, closer respiratory monitoring, timely escalation of intensive care management, and optimization of critical care resources in patients at increased risk of ARDS. Prospective multicenter studies are warranted to validate these findings before routine clinical implementation.

## Figures and Tables

**Table 1:** Comparison of the baseline characteristics, laboratory parameters, and clinical scores of patients across groups.

Variables	Non-ARDS Group (*n* = 154)	ARDS Group (*n* = 48)	t/χ^2^	*p*
Age (years) (mean ± SD)	54.49 ± 11.39	55.83 ± 10.99	−0.67	0.502
Male (*n*/%)	99 (64.3%)	17 (35.4%)	11.32	<0.001 *
Female (*n*/%)	55 (35.7%)	31 (64.6%)		
Diabetes mellitus (*n*/%)	34 (22.1%)	9 (18.8%)	0.08	0.772
Hypertension (*n*/%)	47 (30.5%)	16 (33.3%)	2.15	0.232
Cerebrovascular disease (*n*/%)	10 (6.5%)	7 (14.6%)	-	0.131
Respiratory disease (*n*/%)	20 (13.0%)	16 (33.3%)	9.00	0.003 *
Cardiovascular disease (*n*/%)	18 (11.7%)	9 (18.8%)	1.03	0.311
Renal disease (*n*/%)	24 (15.6%)	11 (22.9%)	0.91	0.340
28-day mortality (*n*/%)	24 (15.6%)	20 (41.7%)	13.12	<0.001 *
BMI <18.5 kg/m^2^ (*n*/%)	10 (6.5%)	9 (18.8%)	11.31	0.010 *
BMI: 18.5–24.9 kg/m^2^ (n/%)	100 (64.9%)	25 (52.1%)		
BMI: 25–30 kg/m^2^ (n/%)	37 (24.0%)	8 (16.7%)		
BMI >30 kg/m^2^ (n/%)	7 (4.5%)	6 (12.5%)		
Creatinine mg/dL (mean ± SD)	1.54 ± 1.08	1.75 ± 1.10	−1.19	0.239
Albumin g/dL (mean ± SD)	2.97 ± 0.52	2.66 ± 0.61	3.15	0.002 *
Total bilirubin mg/dL (mean ± SD)	0.92 ± 0.50	1.16 ± 0.61	−2.46	0.017 *
CRP mg/dL (mean ± SD)	141.77 ± 86.72	166.27 ± 94.19	−1.60	0.113
CAR (mean ± SD)	50.53 ± 33.94	67.87 ± 43.88	−2.51	0.014 *
WBC 10^3^/μL (mean ± SD)	14.32 ± 5.38	16.20 ± 7.63	−1.59	0.118
Hemoglobin g/dL (mean ± SD)	10.48 ± 1.99	10.24 ± 2.10	0.68	0.497
Hematocrit % (mean ± SD)	31.54 ± 5.63	31.02 ± 5.96	0.54	0.594
Platelets 10^3^/μL (mean ± SD)	202.83 ± 95.31	168.23 ± 91.56	2.26	0.026 *
Neutrophils 10^3/^μL (mean ± SD)	11.64 ± 5.08	13.47 ± 7.16	−1.65	0.103
Lymphocytes 10^3/^μL (mean ± SD)	0.85 ± 0.57	0.90 ± 0.60	−0.51	0.610
Monocytes 10^3^/μL (mean ± SD)	0.54 ± 0.30	0.68 ± 1.08	−0.91	0.367
NLR (mean ± SD)	18.07 ± 13.52	23.22 ± 26.62	−1.29	0.203
Lactate mmol/L (mean ± SD)	3.45 ± 2.23	4.47 ± 3.38	−1.96	0.054
APACHE II score (mean ± SD)	19.18 ± 5.24	23.06 ± 5.95	−4.05	<0.001 *
SAPS II (mean ± SD)	34.06 ± 8.08	39.96 ± 10.05	−3.71	<0.001 *
SOFA score (mean ± SD)	5.65 ± 3.27	8.21 ± 3.63	−4.37	<0.001 *
NEWS (mean ± SD)	7.31 ± 3.84	7.10 ± 4.30	0.30	0.766

APACHE, acute physiology and chronic health evaluation; AUC, area under the curve; BMI, body mass index; CAR, C-reactive protein/albumin ratio; CI, confidence interval; CRP, C-reactive protein; NLR, neutrophil-to-lymphocyte ratio; NEWS, national early warning score; OR, odds ratio; ROC, receiver operating characteristic; SAPS, Simplified Acute Physiology Score; SD, standard deviation; SOFA, sequential organ failure assessment; WBC, white blood cell count; ARDS, acute respiratory distress syndrome. Data are presented as mean ± standard deviation or number (%). * *p* < 0.05.

**Table 2:** Independent risk factors for ARDS according to multivariate logistic regression analysis.

Parameter	β	SE	Wald χ^2^	*p*-Value	OR	Lower	Upper
Constant	−2.134	1.374	2.41	0.120	0.12	0.01	1.75
Sex	0.735	0.382	3.71	0.054	2.09	0.99	4.41
Albumin	−0.379	0.353	1.15	0.283	0.68	0.34	1.37
Respiratory disease	0.510	0.456	1.25	0.264	1.67	0.68	4.07
SOFA score	0.122	0.056	4.73	0.030 *	1.13	1.01	1.26

CI, confidence interval; OR, odds ratio; SE, standard error; SOFA, sequential organ failure assessment. * *p* < 0.05

**Table 3:** Diagnostic performance of the ARDS predictive model by ROC curve analysis.

Variables	AUC	Sensitivity	Specificity	Optimal Cutoff Value	95% CI
Albumin	0.654	41.7	85.1	2.40	0.560–0.748
Total bilirubin	0.619	43.8	83.1	1.14	0.517–0.716
CAR	0.627	60.4	72.1	61.11	0.522–0.730
Platelet	0.606	47.9	74.0	137.00	0.507–0.693
Lactate	0.624	43.8	79.2	3.80	0.534–0.713
SOFA score	0.710	54.2	83.1	9.00	0.619–0.794
Combined model	0.727	70.8	72.7	0.220	0.635–0.820

AUC, area under the curve; CI, confidence interval; CAR, C-reactive protein/albumin ratio; SOFA, sequential organ failure assessment.

## Data Availability

The data presented in this study are available on request from the corresponding author due to privacy.

## Abbreviations

The following abbreviations are used in this manuscript:

APACHE	Acute Physiology and Chronic Health Evaluation
AUC	Area Under the Curve
CI	Confidence Interval
ARDS	Acute Respiratory Distress Syndrome
SD	Standard Deviation
OR	Odds Ratio
ROC	Receiver Operating Characteristic
BMI	Body Mass Index
CRP	C-Reactive Protein
CAR	C-Reactive Protein/Albumin Ratio
WBC	White Blood Cell Count
NLR	Neutrophil-to-Lymphocyte Ratio
SAPS	Simplified Acute Physiology Score
SOFA	Sequential Organ Failure Assessment
NEWS	National Early Warning Score
